# Boerhaave Syndrome: Challenges Faced to Achieve Positive Outcomes

**DOI:** 10.7759/cureus.115118

**Published:** 2026-08-24

**Authors:** Nikhil Reddy, Sankar Subramaniam, Chinni Vikram Asokan, Suresh Kumar, Dheeraj Kethireddy

**Affiliations:** 1 Surgical Gastroenterology, Sri Ramachandra Institute of Higher Education and Research, Chennai, IND

**Keywords:** boerhaave syndrome, endoscopic vacuum therapy, oesophageal perforation, oesophagectomy, thoracoscopy

## Abstract

Boerhaave syndrome, the spontaneous barogenic rupture of the oesophagus, is lethal when unrecognised and its cardiorespiratory mimicry frequently delays diagnosis. Optimal management remains debated. We retrospectively reviewed four consecutive patients with spontaneous oesophageal perforation managed over two years at a tertiary surgical-gastroenterology unit. Diagnosis was confirmed by water-soluble oral-contrast computed tomography; presentation, operative findings, complications and outcomes were analysed and compared with the contemporary literature. The four patients were initially assessed by non-surgical services, with an intercostal drain placed for a presumed effusion or pneumothorax before food or purulent fluid in the drain revealed the diagnosis. The perforation was left-sided in three and right-sided in one. Management was individualised: laparoscopy converted to laparotomy with transhiatal primary repair and omental buttress; laparotomy with transhiatal repair and thoracoscopic lavage; a thoraco-abdominal approach; and a right posterolateral thoracotomy. A postoperative leak occurred in two patients and was managed by endoscopic vacuum therapy and by transhiatal oesophagectomy with gastric conduit, respectively. All four patients survived. Food-stained or purulent output from a chest drain placed for a presumed effusion should prompt immediate contrast computed tomography. An individualised, multidisciplinary strategy combining timely source control, thoracoscopic pleural lavage and modern endoscopic rescue achieved survival in every patient, including a rare right-sided rupture.

## Introduction

Boerhaave syndrome is the spontaneous, barogenic, full-thickness rupture of the oesophagus that follows a sudden rise in intraluminal pressure against a closed glottis, classically after vomiting or retching, and typically involves the distal left posterolateral wall. It accounts for approximately 10-15% of oesophageal perforations and, untreated, approaches uniform lethality [1]. Diagnosis is frequently delayed because chest pain, dyspnoea and a pleural effusion mimic myocardial and pulmonary emergencies; patients are therefore often first assessed by cardiologists or pulmonologists, and a chest drain may be placed for a presumed effusion or pneumothorax before the perforation is suspected [1,2]. Time to definitive treatment is the dominant determinant of outcome: a delay beyond 24 hours roughly doubles mortality, whereas early primary repair substantially reduces it [2]. Management spans primary surgical repair, oesophageal resection and, increasingly, endoscopic therapies such as stenting, over-the-scope clipping and endoscopic vacuum therapy (EVT), and is best tailored to the interval, degree of contamination and physiological state [3]. We present four consecutive patients managed over two years at a tertiary surgical-gastroenterology unit, in whom an individualised strategy achieved survival in every case, and we analyse this experience against the contemporary literature.

## Case presentation

We retrospectively reviewed four consecutive patients with spontaneous oesophageal perforation. In every patient, the diagnosis was confirmed by water-soluble oral-contrast computed tomography (CT). Presentation, imaging, operative findings, complications and outcome are summarised for all four cases (Table 1). Written informed consent for publication was obtained. 

**Table 1:** Clinical, radiological, operative and outcome features of the four patients ARDS, acute respiratory distress syndrome; CT, computed tomography; EVT, endoscopic vacuum therapy; F, female; M, male; POD, postoperative day; VATS, video-assisted thoracoscopic surgery.

Feature	Case 1	Case 2	Case 3	Case 4
Age (years) / sex	55 / M	55 / F	35 / M	43 / F
Presenting symptom	Chest pain	Breathlessness	Chest pain, breathlessness	Chest pain
Trigger	Heavy meal, retching	Not specified	Not specified	Evening meal, retching
Initial specialty seen	Cardiology	Emergency	Emergency	Outside hospital
Chest drain finding	Food particles	Food particles	Purulent fluid	Food content
Diagnostic imaging	Oral-contrast CT	Oral-contrast CT	Oral-contrast CT	Oral-contrast CT
Perforation site	Lower oesophagus, left	Distal, left	Distal, left	Right-sided
Operative approach	Laparoscopy → laparotomy, transhiatal	Laparotomy, transhiatal	Thoraco-abdominal	Right posterolateral thoracotomy
Repair / adjunct	Primary repair + omental buttress	Primary repair + VATS lavage	Primary repair	Primary repair
Postoperative complication	ARDS; mediastinal collection	Anastomotic/repair leak	None	Repair leak
Leak management	VATS drainage (POD 12)	Endoscopic clip → EVT	No leak	Oesophagectomy + gastric conduit
Outcome	Survived	Survived	Survived (discharged POD 16)	Survived

Case 1

**Figure 1:**
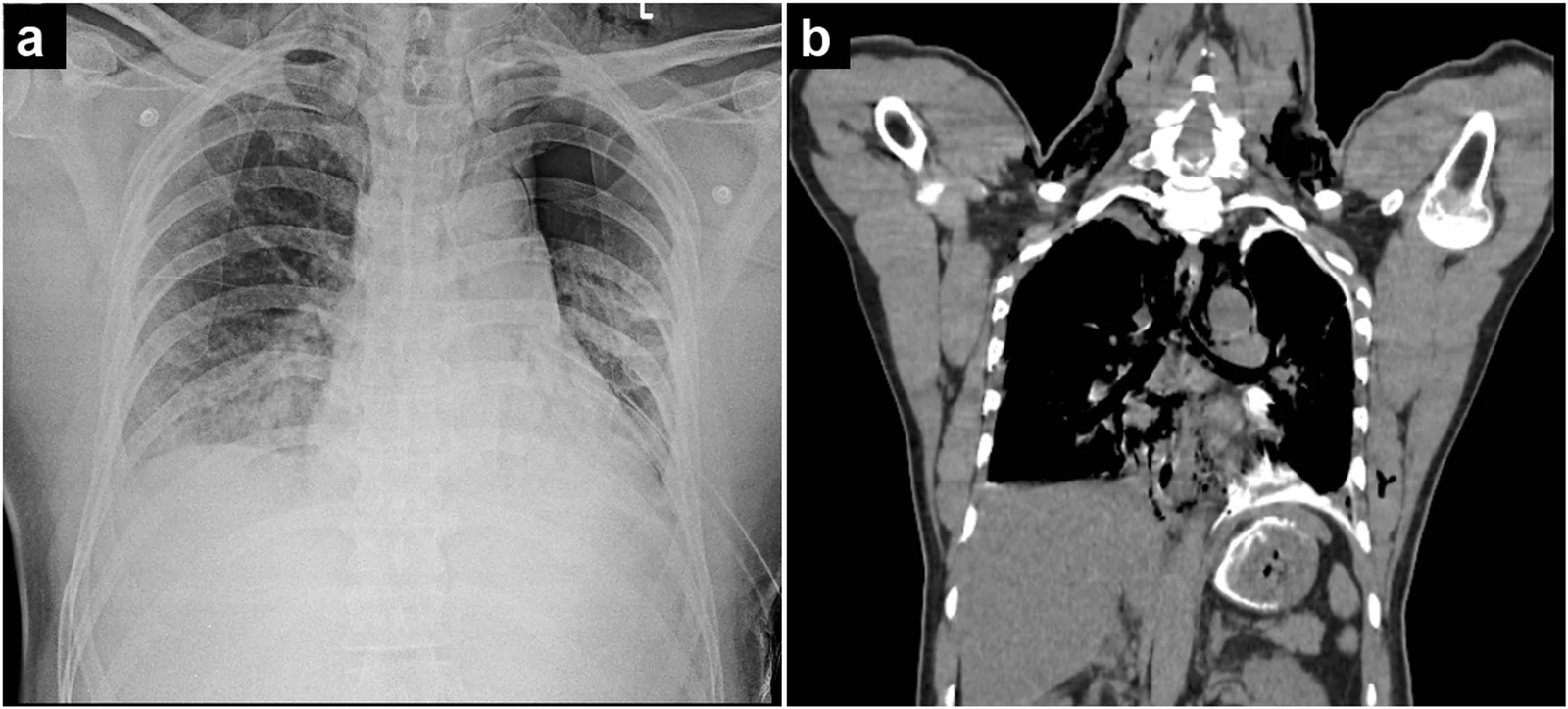
Case 1 Images (a) Chest radiograph showing a left hydropneumothorax with an intercostal drain. (b) Coronal oral-contrast computed tomography showing leak of contrast from the lower oesophagus into the left pleural cavity.

A 55-year-old man presented with chest pain of one day after a heavy meal followed by retching. Cardiac causes were excluded at a cardiology clinic before referral. A chest radiograph showed a left hydropneumothorax with mediastinal shift and lung collapse; an intercostal drain (ICD) was placed and food particles subsequently appeared in it. Oral-contrast CT demonstrated a leak from the lower oesophagus into the left pleural cavity (Figure 1A, 1B). At operation, laparoscopy was converted to laparotomy for technical difficulty; transhiatal mobilisation revealed a left-lateral lower-oesophageal perforation, which was repaired primarily and buttressed with omentum. He developed acute respiratory distress syndrome on postoperative day (POD) 3-4; a contrast study showed no leak but a loculated mediastinal collection, drained by video-assisted thoracoscopic surgery (VATS) on POD 12. After prolonged ventilation and intensive care he recovered.

Case 2

**Figure 2:**
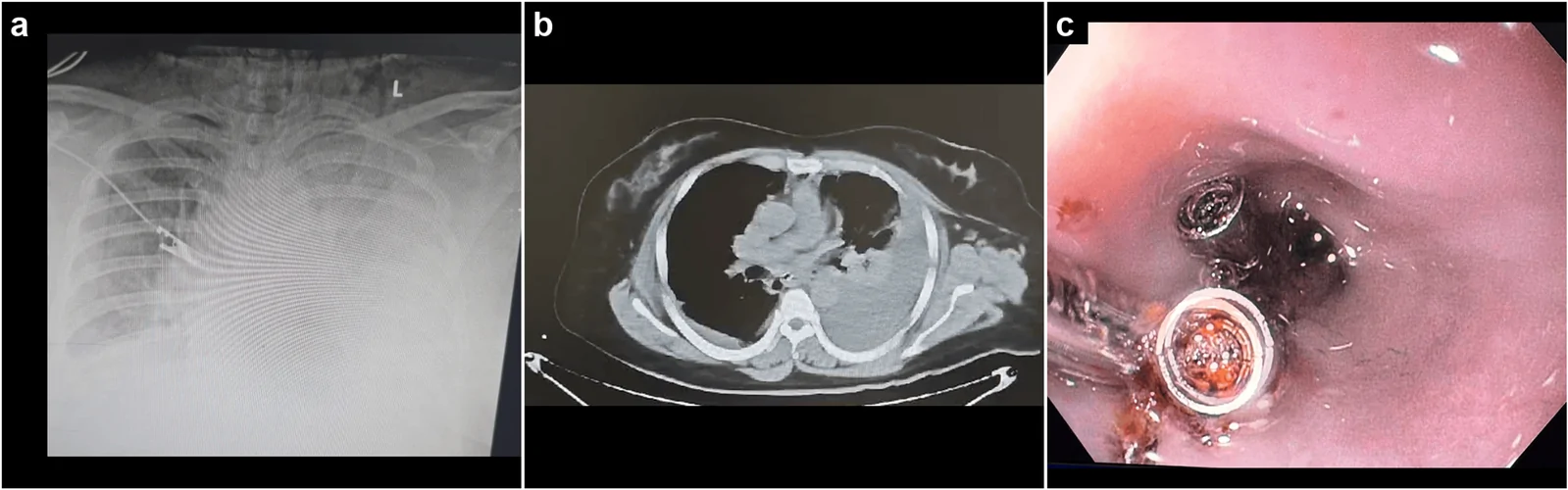
Case 2 Images (a) Chest radiograph showing a massive left pleural effusion. (b) Axial computed tomography showing pneumomediastinum and left pleural effusion. (c) Endoscopic view of the distal-oesophageal perforation with an applied clip.

A 55-year-old woman presented with breathlessness. A chest radiograph showed a massive left pleural effusion; an ICD immediately drained food particles, and oral-contrast CT confirmed a distal oesophageal perforation with pneumomediastinum and effusion (Figure 2A, 2D). She underwent laparotomy with transhiatal repair of the left-lateral perforation and VATS lavage, and was extubated on POD 3. A contrast study on POD 7 showed a leak; endoscopic clipping of the defect was performed (Figure 2C), but a repeat study showed a persistent leak, which was successfully closed with EVT.

Case 3

**Figure 3:**
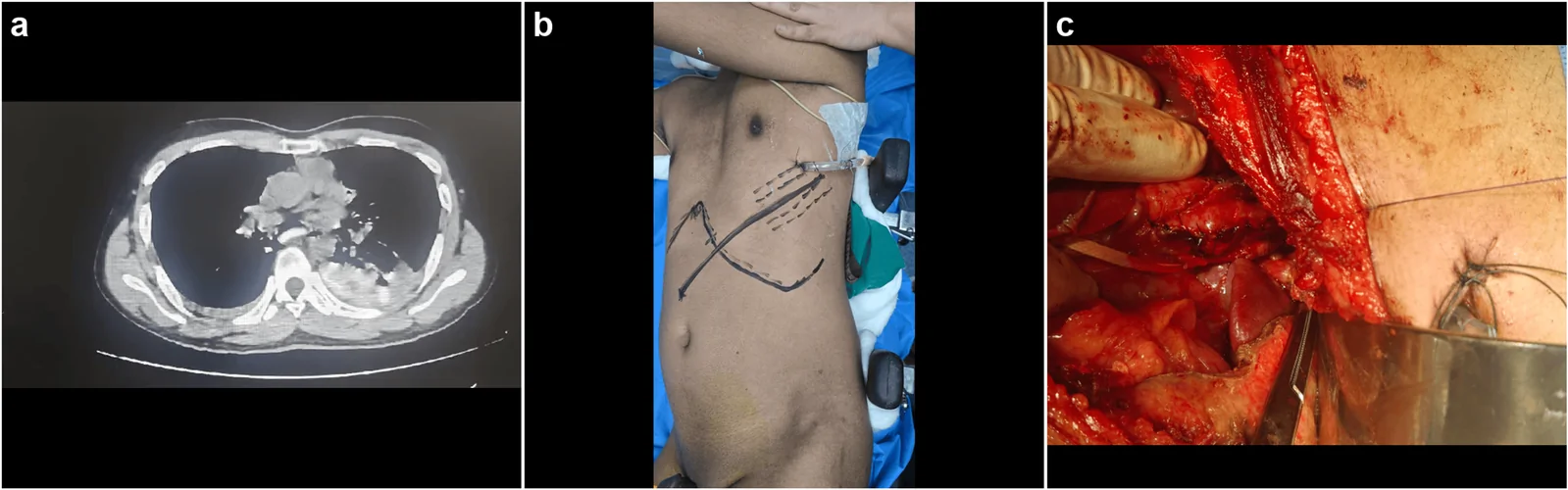
Case 3 Images (a) Axial oral-contrast computed tomography showing leak into the left pleural cavity. (b) Marked thoraco-abdominal incision. (c) Intra-operative view of the repair.

A 35-year-old man presented with chest pain and breathlessness and was found to have a left pleural effusion, for which an ICD was placed. Purulent drain output prompted CT, which showed an oesophageal perforation with contrast leak into the left pleural cavity (Figure 3A). He underwent repair through a thoraco-abdominal incision (Figure 3B, 3C). A postoperative CT confirmed no leak; after seven days in intensive care he was started on oral intake and was discharged on POD 16.

Case 4

**Figure 4:**
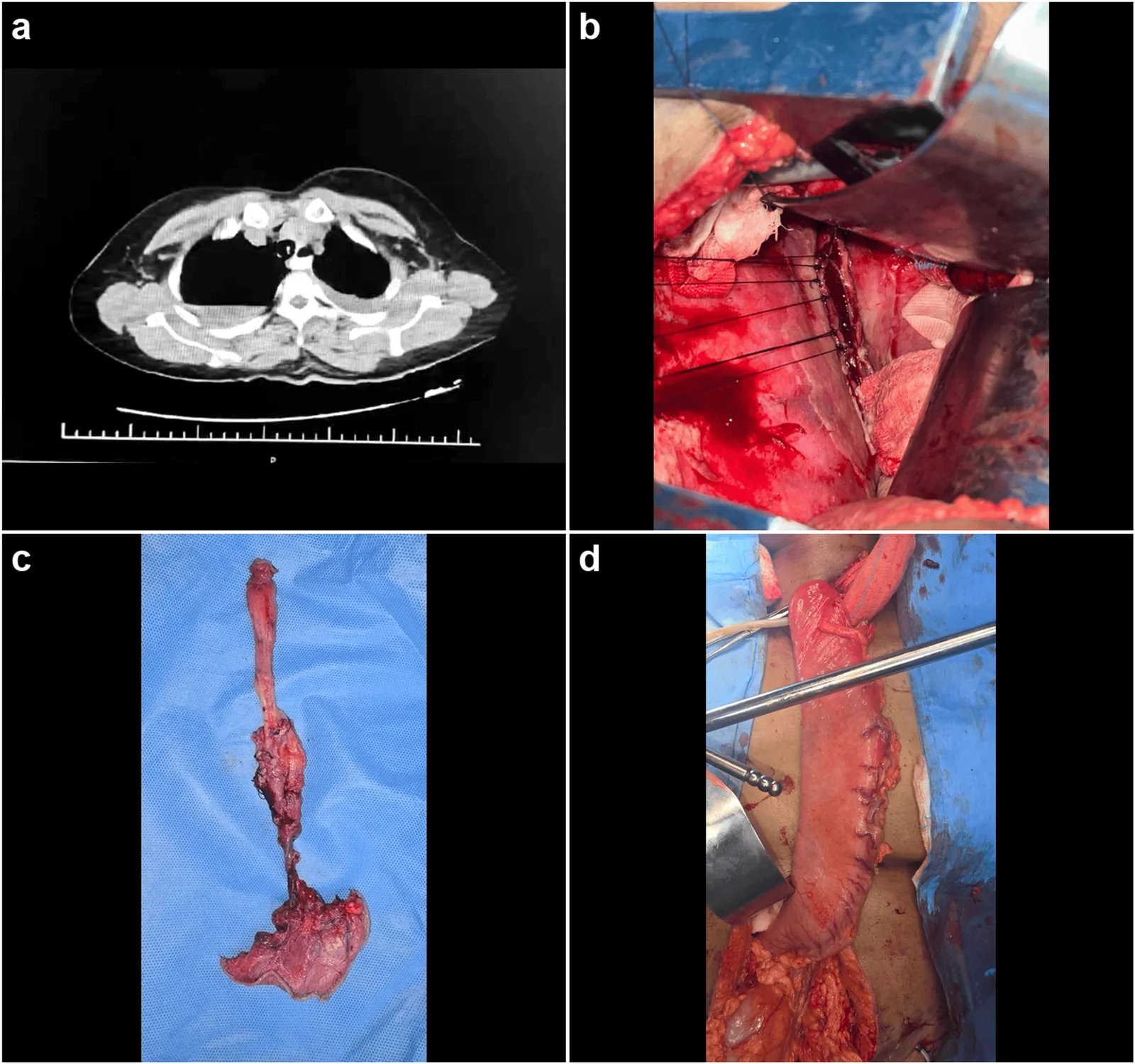
Case 4 Images (a) Axial computed tomography of the thorax after oral water-soluble contrast confirming the perforation. (b) Right posterolateral thoracotomy exposure. (c) Oesophagectomy specimen. (d) Gastric conduit reconstruction.

A 43-year-old woman presented with chest pain of one day after an evening meal followed by retching, having initially received symptomatic treatment elsewhere before referral with worsening pain and breathlessness. Chest radiographs and oral-contrast CT of the thorax confirmed the diagnosis (Figure 4A), and the ICD drained food content. Because the rupture was right-sided, an uncommon location, she underwent a right posterolateral thoracotomy and repair (Figure 4B). On POD 5 she developed tachypnoea and tachycardia, and CT showed a contrast leak; she was salvaged by transhiatal oesophagectomy with gastric conduit reconstruction (Figure 4C, 4D) and recovered.

All four patients survived. The perforation was left-sided in three and right-sided in one; a postoperative leak occurred in two patients and was managed by endoscopic and by resectional salvage, respectively.

## Discussion

Across the four patients (mean age 47 years, range 35 to 55; two men and two women), the diagnosis was established during the index admission by water-soluble oral-contrast computed tomography in every case (4/4, 100%), typically after food-stained or purulent output from an ICD first raised the suspicion; two patients (Cases 1 and 4) presented within about 24 hours of symptom onset, while the other two presented acutely with breathlessness and a pleural effusion. The perforation was distal and left-sided in three patients (75%) and right-sided in one (25%), and the operative approach followed the site of rupture: distal left-sided perforations were repaired transhiatally through the abdomen, a heavily contaminated distal perforation through a thoraco-abdominal incision, and the right-sided rupture through a right posterolateral thoracotomy. All four underwent primary repair (100%), with adjunctive video-assisted thoracoscopic pleural lavage or drainage in three (75%). A postoperative leak occurred in two patients (50%), managed endoscopically (clip followed by EVT) in one and by oesophagectomy with gastric-conduit reconstruction in the other, while a third developed a mediastinal collection without leak. There was no mortality (0%). Case 3 resumed oral intake and was discharged on postoperative day 16.

All four patients survived despite the delayed and misdirected presentations that characterise Boerhaave syndrome. Because chest pain, dyspnoea and a pleural effusion mimic cardiac and pulmonary emergencies, our patients were first evaluated by non-surgical services and a chest drain was placed before the perforation was recognised, typically when food particles or purulent, food-stained fluid appeared in the drain, a simple but decisive bedside sign [4]. This matters because time to definitive treatment is the dominant prognostic factor: mean time to theatre has been reported at 7.3 days in patients who died versus 1.5 days in survivors, and delay beyond 24 hours approximately doubles mortality [2,5]. Contemporary series report mortality of roughly 7-25% [5-7], and meta-analytic data indicate that late presentation need not preclude a good outcome when contamination is controlled [8]. Our 0% mortality is therefore noteworthy.

No single operation suits every patient, and modern practice is severity- and interval-based rather than dogmatic [3]. Our four patients illustrate this spectrum: laparoscopy converted to laparotomy with transhiatal primary repair and omental buttress; laparotomy with transhiatal repair plus VATS lavage; a thoraco-abdominal approach; and a right posterolateral thoracotomy. Laparoscopic transhiatal repair with a gastric or omental buttress and pleural toilet is safe and effective for distal ruptures, with no postoperative leak in a dedicated series [9], and minimally invasive and thoracoscopic strategies have achieved excellent survival in comparably small series [10]. Adjunctive VATS with pleural lavage, used in three of our patients, controls the thoracic sepsis that drives mortality and recurs as a theme in successful series [11].

A postoperative leak, which occurred in two of our patients, is the principal threat after repair and is increasingly managed endoscopically [12]. In Case 2 an early leak was treated first by endoscopic clipping, appropriate for small defects under current guidance [13], and, when this failed, by EVT, which achieved closure. EVT is now a leading rescue for oesophageal transmural defects: dedicated series in Boerhaave syndrome report high closure rates with no mortality [14], and meta-analysis shows higher closure and lower mortality than stenting [15], while stents remain effective when sealing is feasible [8]. At the aggressive end of the spectrum, Case 4 required transhiatal oesophagectomy with gastric-conduit reconstruction for an early leak, an uncommon but life-saving decision when repair fails, albeit one reserved for selected patients because resection carries the highest complication burden [6]. Case 4 was also unusual in being right-sided; across the series we reviewed the perforation was almost invariably distal and left-lateral, so a right-sided rupture is rarely reported and mandates a right thoracotomy and a high index of suspicion.

This is a small, single-centre, retrospective series without a comparator group, and the favourable outcome may reflect selection and the resources of a tertiary unit; the interval to definitive care was not uniformly documented. Nonetheless, the consistency of survival across four physiologically different presentations supports an individualised, multidisciplinary strategy combining timely source control, aggressive thoracoscopic toilet and modern endoscopic rescue.

## Conclusions

Boerhaave syndrome remains lethal when unrecognised, and its cardiopulmonary mimicry continues to delay diagnosis. Food particles or food-stained fluid in a chest drain placed for a presumed effusion should immediately raise the possibility of oesophageal rupture and prompt water-soluble contrast CT. With early source control, a surgical approach tailored to the interval and site, liberal thoracoscopic pleural lavage, and EVT or oesophagectomy to rescue leaks, all four of our patients survived, including one with a rare right-sided rupture. An individualised, multidisciplinary strategy offers the best chance of survival in this dangerous condition.
